# Keeping children safe at home: protocol for three matched case–control studies of modifiable risk factors for falls

**DOI:** 10.1136/injuryprev-2012-040394

**Published:** 2012-06

**Authors:** Denise Kendrick, Asiya Maula, Jane Stewart, Rose Clacy, Frank Coffey, Nicola Cooper, Carol Coupland, Mike Hayes, Elaine McColl, Richard Reading, Alex Sutton, Elizabeth M L Towner, Michael Craig Watson, on behalf of the Keeping Children Safe Study Group

**Affiliations:** 1 Division of Primary Care, Tower Building, University Park, Nottingham, UK; 2 Nottinghamshire Healthcare NHS Trust, Nottingham, UK; 3 Nottingham University Hospitals NHS Trust, Queen's Medical Centre, Nottingham, UK; 4 Department of Health Sciences, University of Leicester, Leicester, UK; 5 Child Accident Prevention Trust, Child Accident Prevention Trust, London, UK; 6 Newcastle Clinical Trials Unit, The Medical School, Newcastle University, Newcastle upon Tyne, UK; 7 Norfolk and Norwich University Hospital, Colney Lane, Norwich, UK; 8 Centre for Child and Adolescent Health, University of the West of England, Bristol, UK; 9 School of Nursing, Nottingham University Hospitals NHS Trust, Queen's Medical Centre, Nottingham, UK

**Keywords:** Falls, case-control study, risk factors

## Abstract

**Background:**

Childhood falls result in considerable morbidity, mortality and health service use. Despite this, little evidence exists on protective factors or effective falls prevention interventions in young children.

**Objectives:**

To estimate ORs for three types of medically attended fall injuries in young children in relation to safety equipment, safety behaviours and hazard reduction and explore differential effects by child and family factors and injury severity.

**Design:**

Three multicentre case–control studies in UK hospitals with validation of parental reported exposures using home observations. Cases are aged 0–4 years with a medically attended fall injury occurring at home, matched on age and sex with community controls. Children attending hospital for other types of injury will serve as unmatched hospital controls. Matched analyses will use conditional logistic regression to adjust for potential confounding variables. Unmatched analyses will use unconditional logistic regression, adjusted for age, sex, deprivation and distance from hospital in addition to other confounders. Each study requires 496 cases and 1984 controls to detect an OR of 0.7, with 80% power, 5% significance level, a correlation between cases and controls of 0.1 and a range of exposure prevalences.

**Main outcome measures:**

Falls on stairs, on one level and from furniture.

**Discussion:**

As the largest in the field to date, these case control studies will adjust for potential confounders, validate measures of exposure and investigate modifiable risk factors for specific falls injury mechanisms. Findings should enhance the evidence base for falls prevention for young children.

## Introduction

Falls are a leading cause of morbidity and mortality in childhood.^1–3^ Globally in 2004, 4.2% of all deaths worldwide in 0–17 year olds were attributed to falls.^4^ Fatal fall rates in those under 20 years of age vary by country, income and gender with the highest rates seen in Eastern Mediterranean and South East Asian regions.^4^ Falls are the 4th leading cause of injury-related death in children in the USA^5^ and the 6th leading cause of injury-related death in children aged 0–14 years in Australia.^6^

Falls are the 13th most common cause of disability-adjusted life years (DALY) lost worldwide.^7^ ^8^ Globally, 50% of the total number of DALYs lost due to falls occur in children younger than 15 years.^4^ In one UK study falls accounted for 60% of traumatic brain injuries in those under 5 years.^9^ In the UK and France they are the most common cause of fatal and serious head injuries^10–12^ and in China they are the leading cause of permanent disability in children aged 0–17 years.^13^ Beyond the age of infancy, children typically use their arms to shield their head; consequently falls result in limb fractures, most commonly of the forearm.^14–18^

In most countries falls are the most common cause of childhood attendance at emergency departments (EDs) accounting for 22% to 52% of attendances.^7^ ^8^ In the UK falls accounted for more than 47 000 hospital admissions in 2009/2010 in children aged 0–14 years.^19^ Falls account for between 25% and 50% of all hospital admissions in low-income and middle-income countries and these rates are thought to be under-reported.^7^ ^20^ The burden placed on healthcare resources is substantial: the annual cost of childhood falls in Canada in 1995 was 630 million Canadian dollars, in Australia in 1994 annual child fall injury costs were estimated at over 130 million Australian dollars and in the USA in 1996 falls were responsible for approximately one-quarter of all childhood unintentional injury-related costs.^6^ ^21^ ^22^

Childhood falls frequently occur from structural components of houses; commonly stairs, balconies and walls.^3^ Under the age of 1, aside from being dropped, falls commonly occur from furniture or car seats; and from steps, staircases or play equipment in 1–3 year olds.^23–27^ Older children aged 5–17 are more likely to fall from objects such as playground equipment.^24^

A systematic review of risk factors for fall-related injuries in children aged >7 years old found few controlled studies. Low socioeconomic status, male sex and young age were consistently found to be risk factors in high-income and low-to-middle-income countries.^7^ Other risk factors included the height of the fall, the surface onto which the child fell and the mechanism of fall.^7^ The review recommended that population-based case–control studies examining risk and protective factors for childhood fall injuries were needed. A more recent systematic review found falls to be associated with lower socioeconomic status, parental education and income, but effects varied across age ranges, gender and mechanism of fall.^28–30^

Case–control studies have examined risk and protective factors for a range of childhood injuries. However, these are often hampered by limitations such as small sample size, use of hospital controls rather than community controls, controlling for a limited range of confounders, the use of non-validated measures of exposure, the use of composite exposure measures precluding estimation of protective effects of individual safety behaviours and a lack of specificity in case definition.^31–36^ Evidence from well designed and executed case–control studies such as those examining the protective effects of cycle helmets^37^ and smoke alarms^38^ has played a highly influential role in policy development and legislation. Similar evidence is now required for potentially modifiable factors for falls prevention in childhood.

The aim of the case–control studies described here is to evaluate the relationship between modifiable risk factors (eg, safety equipment use, behaviours and home hazards) and fall injuries in young children. Limitations of previous studies will be addressed by undertaking separate case–control studies for three common fall mechanisms: falls from furniture, falls down stairs and falls on the same level. Community and hospital controls will be used, a range of confounding factors will be measured and adjusted for and parental reported exposures will be validated by home observations in a sample of cases and controls.

## Methods

### Objectives

The primary objective is to estimate ORs for three types of medically attended falls (either from furniture, down stairs or on one level) occurring in children under the age of 5 years for a range of modifiable risk factors including safety behaviours, safety equipment use and hazards and to adjust for a range of potential confounding factors. Secondary objectives are to explore effects of safety equipment, safety behaviours and hazard reduction by child and family factors which may be associated with differential effectiveness of home safety interventions (child age, gender, ethnicity, single parenthood, housing tenure and unemployment)^39^ and injury severity.

### Study design

Three multicentre matched case–control studies will be undertaken within hospitals in Nottingham, Bristol, Norwich, Newcastle-upon-Tyne, Gateshead, Derby and Great Yarmouth, UK, with recruitment commencing on 14 June 2010.

### Definitions of cases and controls

A fall is defined as: ‘an event which results in a person coming to rest inadvertently on the ground or floor or other lower level’.^40^ Medically attended fall injuries (MAFI) are falls resulting in hospital admission, ED, or Minor Injury Unit (MIU) attendance.

Cases will be defined as children aged 0–4 years attending one of the participating hospitals or MIUs with a MAFI resulting from a stairway fall, fall from furniture or fall on one level that occurred in the home or garden at the address where the child is registered with a general practitioner (GP) (hereafter referred to as the child's home or garden).

Children with intentional or suspected intentional injury, those living in residential care and those who suffered fatal injuries will be excluded. Cases who have a further MAFI 12 months or more after being recruited to one of the studies will be eligible for inclusion as a case if they present with a fall with a different mechanism from the fall that resulted in their initial study recruitment. Cases will be recruited to a maximum of two of the case–control studies. Cases will not be eligible to be subsequently recruited as controls.

Controls will be defined as children aged 0–4 years who did not have a MAFI on the same date as the cases' attendance or admission to hospital. Children living in residential care will be excluded. Controls will be eligible for subsequent recruitment to the three studies as a case, or as a further control if they are recruited 12 months or more after their first recruitment. Controls will not be recruited more than twice to any of the three case–control studies.

### Recruitment of cases and controls

Cases will be approached to take part in the study by clinical staff and recruited by the research team. Cases will be recruited either face-to-face during their medical attendance, by telephone if the parent or guardian have given permission to be contacted via this route or by postal recruitment following their attendance. Telephone contacts or postal invitations will be initiated within 72 h of the medical attendance. One reminder will be sent to people who have not replied to the original postal invitation 2 weeks after the original mailing. Eligibility for the study will be assessed prior to making the approach, based on data recorded in the medical records. Further eligibility assessment will be undertaken when parents return completed questionnaires.

**Figure 1:**
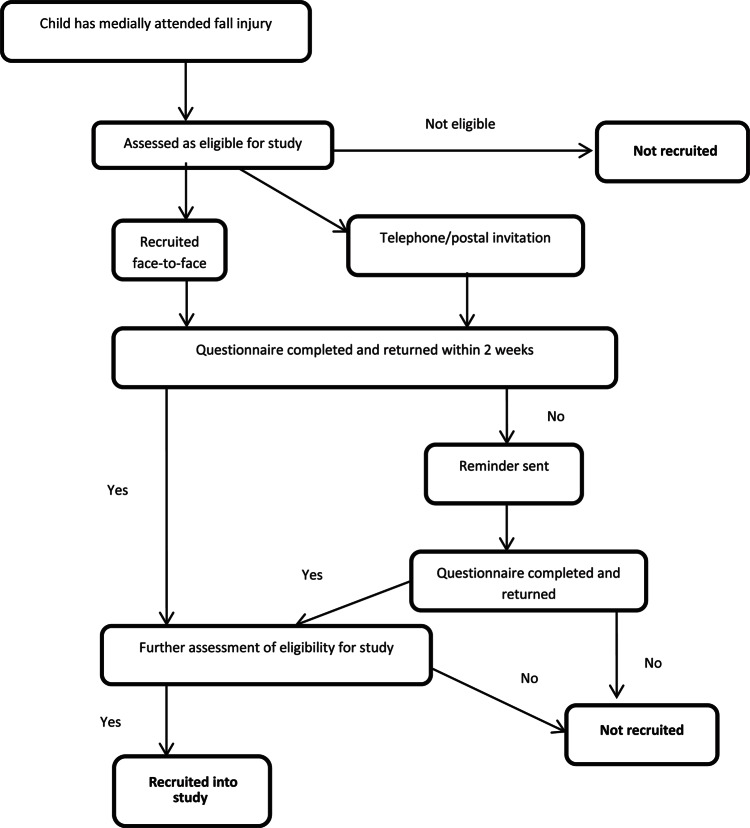
Recruitment of cases.

**Figure 2:**
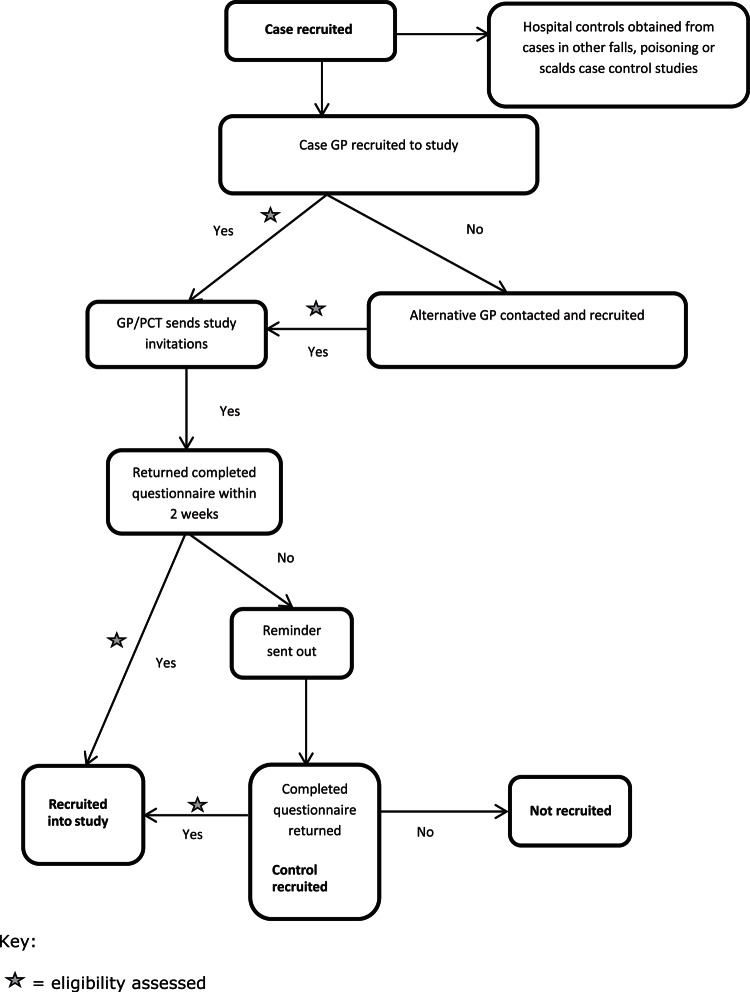
Recruitment of controls.

Two sources of controls will be used; matched community controls and unmatched hospital controls. Community controls will be matched on age (within 4 months of the case's age) and sex and recruited from the register of the cases' GP. In all, 10 children will be identified from the register, with the aim of recruiting an average of 4 for each case and a postal invitation will be sent to the parent/guardian by the GP or the Primary Care Trust. One reminder will be sent to non-responders 2 weeks after the original mailing. Where the practices are unwilling or unable to recruit controls, controls will be recruited from the GP practice geographically closest to the cases' GP practice. Controls will be recruited within 4 months of the case injury. Hospital controls will comprise children with a different mechanism of fall injury (ie, stairway, from furniture or on one level) and children participating in two other ongoing case control studies of poisoning and scalds. All hospital controls recruited up to the date of the last case recruited will be used in the analysis. The process of recruitment of cases and controls is shown in figures 1 and 2.

### Definition of exposures

The time period of interest for assessing exposures is 24 h preceding the MAFI for cases and the 24 h prior to completing the questionnaire for controls, see table 1 for exposures specific to each type of fall.

**Table 1:** Exposures of interest for the three falls case control studies

	Stairway falls	Falls on one level	Falls from furniture
*Safety gates on stairs or used elsewhere† ‡ §	x	x	x
*Baby walkers†	x	x	x
*Play pens†	x	x	x
*Travel cots†	x	x	x
*Stationary activity centres†	x	x	x
Loose carpets on stairs† ‡ §	x		
*Presence of bannisters and *width of bannister gaps† ‡ §	x		
*Presence of handrails† ‡ §	x		
Lighting on stairs† ‡ §	x		
Tripping hazards on stairs† ‡ §	x		
Stairway characteristics (*steepness, width, *landings, *winding stairs, state of repair of steps and *type of stair coverings)† ‡ §	x		
Rugs or carpets firmly fixed to floor† ‡ §		x	
Electric wires or cables trailing across floors† ‡ §		x	
Presence of tripping hazards on the floor† ‡ §		x	
*Use of furniture corner covers† ‡ §		x	
Use of locks on doors leading to gardens or yards† ‡ §		x	
Harnesses in high chairs† ‡ §			x
Bunk beds† ‡ §			x
Leaving child unattended on raised surfaces (not in car seat or bouncing cradle)† ‡ §			x
Placing car seats or bouncing cradles on raised surfaces† ‡ §			x
Frequency of children climbing or playing on furniture† ‡ §			x
Climbing or playing on garden furniture† ‡ §			x
Unsupervised playing in the garden† ‡ §		x	x
Teaching and following safety rules including:^41^			
What to do or not do if the floor is slippery† ‡ §		x	
About running in the house† ‡ §		x	
About jumping on the bed or furniture† ‡ §			x
What to do or how to behave when going down the stairs† ‡ §	x		
About carrying big things or many things while going down stairs† ‡ §	x		

* Exposures validated by home observation.

† Measured in children aged 0–12 months.

‡ Measured in children aged 13–36 months.

§ Measured in children aged 37–59 months.

### Definition of confounding variables

Potential confounding variables to be measured include: sociodemographic and economic characteristics (eg, family size and structure, ethnicity, overcrowding, housing tenure, receipt of state-provided means-tested benefits, maternal age) and validated measures of child behaviour and temperament (Infant, Early Child and Child Behaviour Questionnaires (activity and high intensity pleasure subscales)),^42–46^ parenting daily hassles (parenting tasks subscale),^47^ ^48^ mental health (Hospital Anxiety and Depression Scale (HADS)),^49^ general health visual analogue scale,^50^ paediatric quality of life^51^ ^52^ and time cared for outside the home and place of out-of-home care.

### Measurement of exposures and confounding variables

Three age-specific questionnaires for completion by parents will be used (age 0–12 months, 13–36 months and 37–59 months), containing previously validated questions wherever possible. Case questionnaires were piloted with families in the paediatric ED in Nottingham University Hospital and control questionnaires were piloted with parents attending children's centres in Nottingham to assess face validity, comprehension, ease and time to completion. To increase response rates, participants returning a completed questionnaire will be sent a £5.00 (equivalent to US$7.70 or €6.00) gift voucher for use in local shops.^53^

### Validation of exposure measurement

Parental reported exposures will be validated by home visits to a sample of cases and controls to assess agreement between reported and observed safety behaviours, safety equipment use and home hazards. All parents or guardians participating in the case–control studies will be asked if they are willing to participate in other child safety research projects, one of which is the validation study. Those agreeing will be visited at home as soon as possible after study questionnaires are returned. Home visits will be conducted by trained researchers using a standardised checklist. Participants will be asked if they have made any changes to safety behaviours, safety equipment use or hazards over the preceding 3 months and if changes have been made, what the safety behaviour, safety equipment use or presence of hazards was prior to implementation of change. Researchers conducting home visits will be blind to respondents' questionnaire responses in the case–control study.

## Analysis

### Validation of exposure measures

The sensitivity, specificity, positive and negative predictive values (and 95% CIs) will be estimated for each safety behaviour, safety equipment used and hazard comparing observations at the validation visit with questionnaire responses.

### Case–control studies

Causal diagrams will be drawn for each falls mechanism to describe interrelationships between variables and distinguish confounding variables and likely effect mediators prior to undertaking analysis. Exposure and confounding variables will be described separately for cases and controls using frequencies and percentages for categorical variables and means (and SDs) or medians (and IQR) for continuous variables, dependent on their distributions.

The analysis of cases and matched controls will use conditional logistic regression to estimate ORs and 95% CI for each fall mechanism, unadjusted and adjusted for confounding variables identified from causal diagrams. Analyses will not be adjusted for effect mediators (ie, variables potentially in the causal chain between exposure and outcome). Where variables are identified as effect modifiers, stratified results will be presented in addition to adjusted results.

The analysis of cases and hospital controls (cases with other injury mechanisms) will use unconditional logistic regression to estimate unadjusted and adjusted ORs for each case control study. As controls are not individually matched to the cases, the analysis will adjust for age, sex, deprivation (measured using the Index of Multiple Deprivation 2010 at super output area level)^54^ and distance from hospital, in addition to other potential confounding variables, which will be included in the models under the conditions discussed above. Where we hypothesise that exposures may be associated with multiple injury mechanisms we will categorise the control groups by injury mechanism and estimate separate ORs comparing cases with each of these control groups.

Differential effects by child and family factors and injury severity will be examined by adding interaction terms to logistic regression models.

### Sample size

#### Validation of exposure measures

A total of 80 participants will allow estimation of 95% CI of ±20% around a sensitivity of 80%, assuming a minimum of 20% of participants observed to have the safety behaviour, safety equipment use or hazard of interest. As it is plausible that sensitivity will vary between cases and controls, 80 cases and 80 controls will be recruited across participating hospitals and MIUs.

#### Case–control studies

To detect an OR of 0.7, based on 80% power, 5% significance level, a correlation between matched cases and controls of 0.1, an average of 4 controls per case and a range of exposure prevalences from previous research (use of stairgates (45%), baby walkers (36%), playpens (42%), stationary activity centres (24%), stairgates across doors (30%), rugs firmly fixed to floors (54%), floors clear of tripping hazards (43%) and not leaving child unattended on raised surfaces (65%)),^55^ ^56^ each case–control study will require 496 cases and 1984 matched controls.

## Ethics committee and regulatory approvals

Ethical approval has been provided by the Nottingham 1 Ethics Committee (reference number: 09/H0407/14). Approval has been obtained from National Health Service Research & Development Departments providing research governance to participating hospitals and MIUs.

## Discussion

These three falls case control studies will be the largest in the field to date. As they will adjust for a range of potential confounders, validate measures of exposure, investigate modifiable risk factors for specific injury mechanisms and use community and hospital controls, they should address some of the deficiencies of previous observational studies. However, the potential for bias still exists. Selection bias may occur through non-response of cases and controls. Attempts to minimise this include using a piloted questionnaire containing standardised and validated tools, use of incentives for questionnaire completion and reminders. Misclassification bias can arise when participants are incorrectly classified as having or not having an exposure of interest. As our exposures are self-reported and as some responses may be considered by parents as being more socially desirable or acceptable than others, it is possible that parents may over report safety behaviours and safety equipment use and under report hazards. In addition, parental recall of some exposures may be inaccurate. Misclassification occurring differentially between cases and controls can result in biased estimates of effect and not always towards the null value.^55^ Efforts to minimise misclassification bias include the use of non-judgemental language in the study documentation, time period restrictions for measuring exposures limited to 24 h prior to injury for cases or 24 h prior to questionnaire completion for controls, initiating study invitations within 72 h of the medically attended fall injury and validating exposures in a sample of cases and controls. These multicentre case–control studies should provide evidence on the effect of potentially modifiable risk factors for fall injuries in young children that can be used to develop appropriate public health interventions.

## Appendix 1

### The Keeping Children Safe Study Group

Bristol: Toity Deave^1^, Trudy Goodenough^1^, Bryony Kay^2^

Child Accident Prevention Trust: Mike Hayes^3^

Derby: Joanne Culshaw^4^, Susie Hewitt^4^, Liz Richardson^4^, Rosemary Smith^4^

Great Yarmouth: Donna Wade^5^, Patricia Hagan^5^, Patricia Barclay^5^

Newcastle: Adrian Hawkins^6^, Paul Hindmarch^6^

Norwich: Gosia Majsak-Newman^7^, Clare Ferns^7^, Nathalie Horncastle^8^, Emma Rayfield^9^, Barbara Stewart^9^

Nottingham: Joanne Ablewhite^10^, Isobel Allwood^11^, Natasha Barnes^11^, Penny Benford^10^, Clare Bryan^12^, Philip Miller^11^, Lindsey Pearson^11^, Persephone Wynn^10^, Ben Young^10^

^1^Centre for Child & Adolescent Health, University of the West of England, Bristol, Oakfield House, Oakfield Grove, Bristol BS8 2BN, UK

^2^Emergency Department, United Bristol Healthcare Trust, Bristol Royal Infirmary, Upper Maudlin Street, Bristol, BS2 8HW, UK

^3^Child Accident Prevention Trust, Child Accident Prevention Trust, Canterbury Court (1.09), 1–3 Brixton Road, London SW9 6DE, UK

^4^Derby Hospitals NHS Foundation Trust, Royal Derby Hospital, Uttoxeter Road, Derby DE22 3NE, UK

^5^James Paget University Hospital, Lowestoft Road, Gorleston, Great Yarmouth, Norfolk, NR31 6LA, UK

^6^Newcastle Upon Tyne Hopsitals Foundation Trust, Great North Childrens Hospital, Research Unit, Level 2, New Victoria Wing, Royal Victoria Infirmary, Queen Victoria Road, Newcastle-upon-Tyne, NE1 4LP, UK

^7^Norfolk and Norwich University Hospitals NHS Trust, Colney Lane, Norwich, NR4 7UY, UK

^8^Faculty of Medicine and Health Sciences, University of East Anglia, Norwich NR4 7TJ, UK

^9^Norfolk, Great Yarmouth and Waveney Primary Care Research Network, NHS Norfolk, Lakeside 400, Broadland Business Park, Norwich, NR7 0WG, UK

^10^Division of Primary Care, Tower Building, University Park, Nottingham, NG7 2RD, UK

^11^Nottingham University Hospitals NHS Trust, Queen's Medical Centre, Nottingham, NG7 2UH, UK

^12^Nottinghamshire Healthcare Trust, Nottinghamshire Healthcare NHS Trust, 2nd Floor, Duncan Macmillan House, Porchester Road, Mapperley, Nottingham NG3 6AA, UK
